# OPA1: 516 unique variants and 831 patients registered in an updated centralized Variome database

**DOI:** 10.1186/s13023-019-1187-1

**Published:** 2019-09-10

**Authors:** Bastien Le Roux, Guy Lenaers, Xavier Zanlonghi, Patrizia Amati-Bonneau, Floris Chabrun, Thomas Foulonneau, Angélique Caignard, Stéphanie Leruez, Philippe Gohier, Vincent Procaccio, Dan Milea, Johan T. den Dunnen, Pascal Reynier, Marc Ferré

**Affiliations:** 1 Département d’Ophtalmologie, Centre Hospitalier Universitaire d’Angers, Angers, France; 2 Unité Mixte de Recherche MITOVASC, CNRS 6015/INSERM 1083, Université d’Angers, Angers, France; 3 Centre de Compétence Maladie Rare, Clinique Jules Verne, Nantes, France; 4 Département de Biochimie et Génétique, Centre Hospitalier Universitaire d’Angers, Angers, France; 5 Singapore National Eye Center, Singapore Eye Research Institute, Duke-NUS, Singapore, Singapore; 6 Human Genetics and Clinical Genetics, Leiden University Medical Center, Leiden, The Netherlands

**Keywords:** OPA1, Dominant optic atrophy, Neurological disorders, Database, Sequence variant, Interoperability

## Abstract

**Background:**

The dysfunction of OPA1, a dynamin GTPase involved in mitochondrial fusion, is responsible for a large spectrum of neurological disorders, each of which includes optic neuropathy. The database dedicated to OPA1 (*https://www.lovd.nl/OPA1*), created in 2005*,* has now evolved towards a centralized and more reliable database using the Global Variome shared Leiden Open-source Variation Database (LOVD) installation.

**Results:**

The updated *OPA1* database, which registers all the patients from our center as well as those reported in the literature, now covers a total of 831 patients: 697 with isolated dominant optic atrophy (DOA), 47 with DOA “plus”, and 83 with asymptomatic or unclassified DOA. It comprises 516 unique *OPA1* variants, of which more than 80% (414) are considered pathogenic. Full clinical data for 118 patients are documented using the Human Phenotype Ontology, a standard vocabulary for referencing phenotypic abnormalities. Contributors may now make online submissions of phenotypes related to *OPA1* mutations, giving clinical and molecular descriptions together with detailed ophthalmological and neurological data, according to an international thesaurus.

**Conclusions:**

The evolution of the *OPA1* database towards the LOVD, using unified nomenclature, should ensure its interoperability with other databases and prove useful for molecular diagnoses based on gene-panel sequencing, large-scale mutation statistics, and genotype-phenotype correlations.

**Electronic supplementary material:**

The online version of this article (10.1186/s13023-019-1187-1) contains supplementary material, which is available to authorized users.

## Background

The commonest form of inherited optic neuropathy, called dominant optic atrophy (DOA) or optic atrophy-1 (OPA1; MIM# 165500), was initially described by Kjer [1]. The frequency of the disease is estimated at 1/30,000 worldwide [2], although a higher incidence of 1/10,000 was reported in Denmark, probably due to a founder effect [3, 4]. The disease, generally diagnosed in early childhood, is characterized by a progressive bilateral loss of visual acuity, centrocecal, central or paracentral visual field defects, temporal or diffuse optic nerve pallor with optic disc excavation, and blue-yellow dyschromatopsia or generalized color vision deficits [5, 6]. DOA is associated with a marked intra- and inter-familial clinical variability and incomplete penetrance, estimated at about 90% in the familial forms of the disease [7].

Mutations in the optic atrophy 1 gene (*OPA1*; MIM# 605290), located on chromosome 3q28-q29, were first reported in 2000 [8, 9]. The *OPA1* gene is responsible for about 60–80% of the cases of DOA with a genetic diagnosis [8–10]. *OPA1*, which has 30 coding exons, including three alternative exons [11], is transcribed in 8 alternative splicing variants, encoding 8 isoforms of 907–1015 amino acids of a mitochondrial dynamin-related GTPase, ubiquitously expressed and anchored to the mitochondrial inner membrane, that play a key role in the fusion of the mitochondrial network [12–14].

Since 2003, phenotype-genotype studies have led to the identification of syndromic DOA phenotypes, the so-called DOA “*plus*” (DOA+; MIM# 125250) syndromes, mainly occurring in young adults and associating *OPA1* variants with optic atrophy and sensorineural deafness [15, 16], ataxia, myopathy, peripheral neuropathy, and progressive external ophthalmoplegia [17–22] in up to 20% of the patients [23]. Since 2011, a new, early-onset OPA1-related syndromic entity, distinct from those previously described, has been reported in some patients with a severe neurological syndrome associating early-onset optic neuropathy with spinocerebellar degeneration, pyramidal signs, peripheral neuropathy, gastrointestinal dysmobility and retarded development, a phenotype fully compatible with the Behr syndrome (MIM# 210000) [24–27]. Other rare associations of *OPA1* mutations have been reported with spastic paraplegia [23], the multiple sclerosis-like syndrome [28], severe syndromic cardiomyopathy [29], and syndromic parkinsonism and dementia [30, 31].

In cases of isolated DOA, most of the variants result in the loss of function of the mutated allele, supporting the notion that haploinsufficiency is the main pathological mechanism of the disease [32]. Conversely, patients with DOA+ syndromes often carry a missense variant rather than a truncating *OPA1* mutation, suggesting that the risk of developing syndromic DOA is significantly greater in the case of a missense mutation in the GTPase domain than for a truncating mutation [2]. In this respect, a recurrent missense variant was identified as a strong contributor to the DOA*+* syndrome, i.e. the c.1499G > A mutation leading to a p.(Arg500His) change in the GTPase domain [10]. A bi-allelic mode of inheritance has been recently shown to be the main cause of the early-onset Behr phenotype, associating a pathogenic variant with a hypomorphic variant. These complex genotype-phenotype correlations in *OPA1* mutation carriers should lead to the reclassification of related disorders, thereby contributing to improved genetic counseling. This underscores the importance of the *OPA1* locus-specific database associating genetic and clinical data, which should facilitate the identification of new genotype-phenotype correlations in *OPA1*-related disorders.

Although to date more than 500 unique *OPA1* variants, mostly family-specific, have been reported (see *https://www.lovd.nl/OPA1*), the original *e*OPA1 database, published in 2005, contained only variants and references gathered from publications [33]. The study of over a thousand patients diagnosed in our clinical laboratory [34] led us to transform our restricted molecular *OPA1* database into a clinico-biological database for DOA, aimed at collecting patient data with a full record of clinical, electrophysiological and biochemical data [35]. Today, the extensive molecular diagnosis made possible by high-throughput sequencing (HTS) allows us to migrate to a common installation integrating all known human genes, i.e. to evolve towards a central database. We here describe the evolution of this database into a new central database, indicating the procedure for data submission, the benefits for the users, and full data analysis.

## Results and discussion

**Fig. 1:**
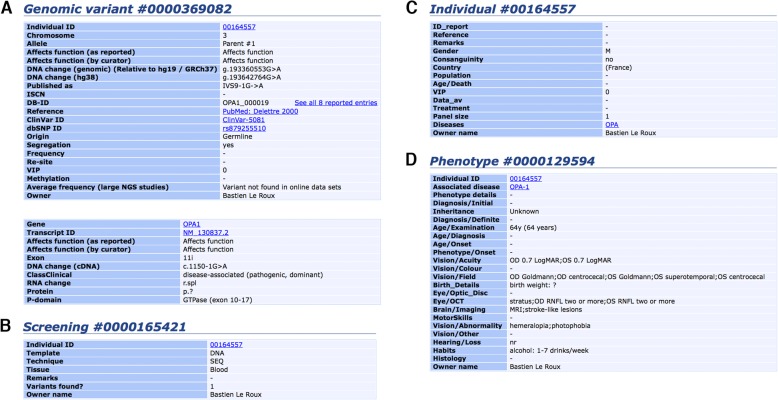
Sample recording for a given patient in the *OPA1* database. **a**. molecular items (“Variant remarks” line removed to save space); **b**. screening items; **c**. individual items; and **d**. phenotype items. Abbreviations and legends of the fields are given by following the link “Legend” on the web page of each table; “SEQ”: sequencing (Sanger); “M”: male; “(France)”: reported by the laboratory in France; “OD”: *oculus dexter* (right eye); “OS”: *oculus sinister* (left eye); “0.7 LogMAR”: best corrected visual acuity 0.7 LogMAR (HP:0030560). “centrocecal”: centrocecal scotoma (HP:0000576); “RNFL two or more”: mean retinal nerve fiber layer thinning in 2 or more quadrants; “MRI”: brain MRI performed; “hemeralopia”: hemeralopia (HP:0012047); “photophobia”: photophobia (HP:0000613). Data as of October 12, 2018

The *OPA1* database contains four main independent but interconnected tables labelled “Variants”, “Screening”, “Individual” and “Phenotype”. These tables are visible on a typical web page entry as shown in Fig. 1. The “Variants” table includes information about the sequence variations at the genomic (DNA) and the transcript variant (cDNA) levels, for both transcripts 8 and 1 (not shown), as well as the reported and concluded status for each variant (Fig. 1a). The “Screening” table gives details of the methods and techniques used for investigating the structural variants and the tissue analyzed (Fig. 1b). The “Individual” table contains details of the patient examined, including gender, geographic origin, and patient identification, if applicable, as listed in the original manuscript (Fig. 1c). The “Phenotype” table indicates the phenotypic features including the visual acuity and field, the OCT report, and the results of brain imaging (Fig. 1d).

### Molecular relevance

**Fig. 2:**
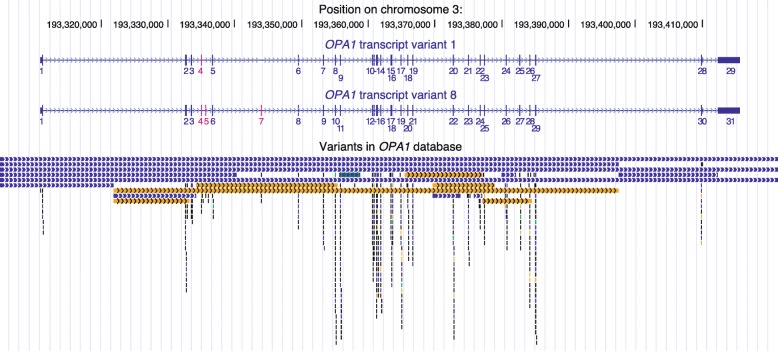
Distribution of the 516 unique genomic variants in the LOVD *OPA1* database (compact view). Eighteen large rearrangements (eleven deletions, six duplications, and one deletion-insertion) are shown as extended bars with rafters, substitutions as black bars, deletions as blue bars, insertions as green bars, and duplications as orange bars. At the top are reported the genomic coordinates on human chromosome 3 (assembly GRCh37/hg19), and *OPA1* transcript variants 1 and 8 structure in navy blue with alternative exons in pink, including exon numbering. The full view detailing the names of each mutation is available in Additional file 2. Adapted from UCSC Genome Browser (*http://genome.ucsc.edu*) with the LOVD *OPA1* database custom track; data as of October 12, 2018

**Fig. 3:**
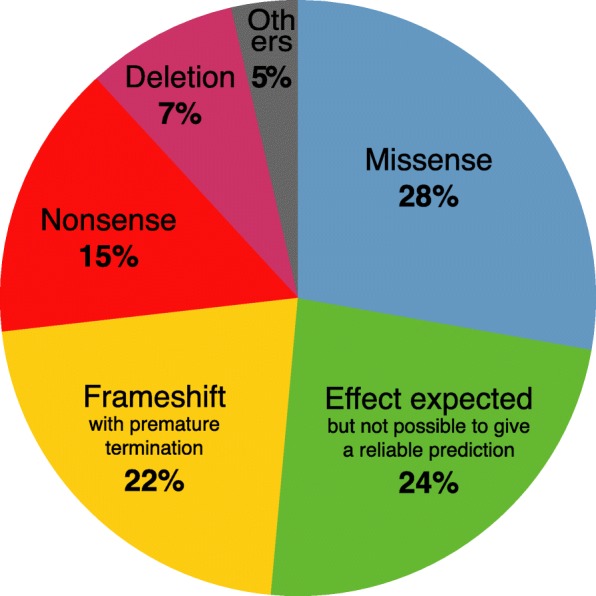
Distribution of the different effects on the protein of the *OPA1* variants considered pathogenic. Other consequences (5%) include: synonymous (11), no protein is produced (5), duplication (3), and extension (1). Data as of October 12, 2018

To date, the database contains 516 unique variants, of which 80% (414) are considered pathogenic sequence variants. These variants, which mainly affect the coding sequence and exon-intron boundaries of the gene, are mainly located in the GTPase and dynamin domains of the protein (exons 10 to 26), highlighting the importance of these domains in OPA1 functions (Fig. 2). Among the most frequently observed pathogenic *OPA1* variants, 28% are missense variants; 24% are associated with altered splicing, which produces effects that are difficult to predict reliably; 22% are frameshift variants; 15% are nonsense variations; and 7% are deletions (Fig. 3). Interestingly, 149 of the unique variants in the database (29%) are unpublished in the literature, i.e. have been submitted to our database only (Additional file 1).

Although only a few mutations are recurrent, some have been frequently reported, for instance, the c.2873_2876del variant in exon 29, which induces a p.(Val958Glyfs*3) frameshift mutation leading to a premature protein truncation, has been reported 22 times; the c.1311A > G variant in exon 14, which induces a missense mutation p.(Ile437Met) that is considered asymptomatic by itself, as well as the c.2635C > T variant in exon 26, which induces a nonsense mutation p.(Arg879*), have been reported 16 times each.

Recently, the Global Variome shared LOVD server has integrated the data from The Genome Aggregation Database (gnomAD), which is the aggregation of the high-quality exome (protein-coding region) DNA sequence data for tens of thousands of individuals [36]. However it was decided not to add these variants as a new record, but only to indicate the frequency reported in gnomAD for each variant present in the server, in order not to flood the LSDBs with data not related to a phenotype. This information is particularly useful at the time of the curation, as well as to assess its relevance. In total, 7% of the unique variants (36) in our database are listed with a frequency in gnomAD. Interestingly, we have assigned a “(probably) non-pathogenic” or “variant of unknown significance” status to all variants with a frequency greater than 0.001% in gnomAD; all the variants we labelled as pathogenic have a very rare frequency in gnomAD, at most nearly 0.001% (13 out of about 13,000 alleles) for the c.239A > G variant in exon 2, which induces a p.(Tyr80Cys) missense mutation. This last-mentioned variant has been reported twice independently as pathogenic in our database, which is a strong argument for concluding to the pathogenicity of a missense mutation; conversely, it is listed without clinical significance in the NCBI dbSNP (Build 151, dbSNP# rs151103940) [37], highlighting the increased accuracy of the LSDB approach as it applies to our database.

### Clinico-ophthalmological relevance

To date, the database includes 831 patients (182 males, 131 females, and 518 patients of unspecified gender). Among these, 697 patients had isolated DOA, 47 had DOA+ (including 12 with hearing loss), and 83 were asymptomatic or unclassified. In addition, four of the patients were reported with phenotypes that are not referenced as being associated with *OPA1*, i.e. ocular albinism type I (OA1; MIM# 300500); polyneuropathy, hearing loss, ataxia, retinitis pigmentosa, and cataract (PHARC; MIM# 612674); spinocerebellar ataxia-5 (SCA5; MIM# 600224); and autosomal recessive spastic paraplegia-18 (SPG18, MIM# 611225).

The database includes a new set of full clinical data for 88 patients consulting at our Ophthalmological Center, in addition to the 30 patients already described in 2015 [35], as well as 60 patients from our Molecular Genetics Laboratory, now representing all the data (178 patients) available from our Center, along with data from 232 patients, retrieved by the curator from publications. In particular, all the published data from the research teams of our European network on inherited optic neuropathies (France, Germany, Italy, United Kingdom) have been integrated; their unpublished data, and those of any other team that may emerge, will be gradually integrated. Overall, since the last major update in 2015 [35], the number of patients in our database has more than doubled, increasing from 328 to 831, with a larger proportion of patients for whom full clinical data is now available, increasing from about 10 % to almost half. Interestingly, 30% of the patients in the database are unpublished in the literature, i.e. have been submitted to our database only, 178 (two thirds) submitted by our Center, France, and 74 (a third) from abroad, outside France (six independent submitters from Germany, Netherlands, United Kingdom and the USA).

The ophthalmological information recorded includes the age at which the patient was examined (i.e. the age of the patient to whom the registered phenotype refers), the best corrected visual acuity, the visual field parameters, the mean thickness of the retinal nerve fiber layer (RNFL) and the ganglion cell layer (GCL), as measured by optical coherence tomography (OCT), together with the name of the manufacturer of the OCT apparatus. Visual acuity is expressed using the logarithm of the minimum angle of resolution (LogMAR) chart, the de facto standard in vision research.

### Central database relevance

The majority of databases, which are central, encompass all the genes of an organism, as in sequence databases [38, 39] or in databases oriented towards non-pathogenic variations [36, 37]. In contrast, databases reporting pathogenic variations, i.e. the so-called locus-, gene- or disease-specific databases (LSDB, GSDB or DSDB), have proved to be the most complete [40] since they benefit from the participation of a curator who is a referent specialist for the gene or disease considered. Unfortunately, these databases are often based on isolated initiatives, using various interfaces hosted on different servers, rendering their interoperability and intuitive use rather difficult. Therefore, the Human Variome Project currently favors the centralization of LSDBs at *https://databases.lovd.nl/shared* [41, 42].

**Fig. 4:**
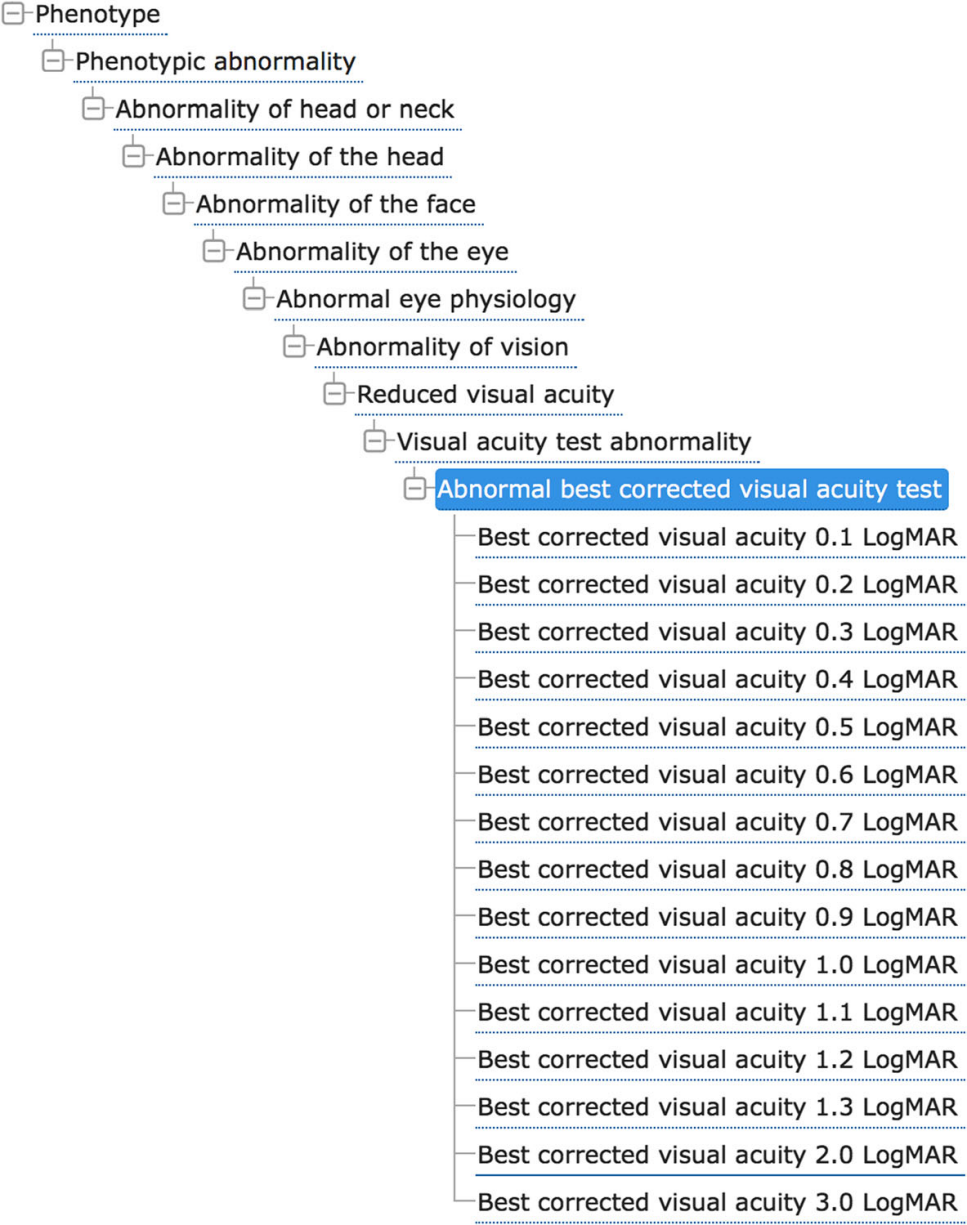
Tree view of the Human Phenotype Ontology term “Abnormal best corrected visual acuity test” (HP:0030532). In the Ontology Lookup Service [44]. The term is highlighted, superclasses indicated above, subclasses indicated below. Data as of Human Phenotype Ontology (HPO) version 2018-06-13

This centralization is the major asset of the work reported here, along with the exhaustive inclusion of patients from our ophthalmological center and data collected from the literature as described above. The implementation of phenotypic descriptions of all patients from the database using the Human Phenotype Ontology (HPO) [43] offers a standard vocabulary for referencing phenotypic abnormalities. Figure 4 shows an example of the hierarchy of terms used for visual acuity. Genomic medicine calls for the precise definition of phenotypic variations [45–47] and descriptions of human disease using HPO annotations are key elements in several algorithms designed for molecular diagnosis and genetic research. The HPO description of the results of ophthalmological examinations have become mature enough to be used in our database [43], although the definition of some terms, now under discussion with the HPO, will be included in the *OPA1* database after validation.

### Ongoing developments

Some *OPA1* patients, already referenced, carry a second mutation in another gene, which it is now technically possible to include in the databank. This would allow the inclusion of other genes involved in neurological diseases affecting mitochondrial dynamics and bioenergetics. For instance, peripheral neuropathy has been linked to *OPA1* mutations, and optic neuropathy to *MFN2* (MIM# 608507) mutations, thus revealing the close proximity of the diseases [48]. Thus, we will integrate *MFN2*, responsible for Charcot-Marie-Tooth neuropathy type 2A (MIM# 609260, 617,087) [49], as well as genes that our team has been involved with recently, i.e. *ACO2* (MIM # 616289) responsible for optic atrophy-9 (OPA9; MIM# 616289) [50], *DNM1L* (MIM #603850) responsible for optic atrophy-5 (OPA5; MIM# 610708) [51], *RTN4IP1* (MIM# 610502) responsible for optic atrophy-10 (OPA10; MIM# 616732) [52], as well as *AFG3L2* (MIM# 604581) and *SPG7* (MIM# 602783), which we recently found associated with optic atrophy in addition to the other neurological symptoms already reported [53].

## Conclusion

The integration of the *OPA1* database into the central LOVD database means that *OPA1* shares a common platform with 22,981 other human genes as referenced to date in *databases.lovd.nl/shared*. This major step constitutes a computational bridge between genome biology and clinical medicine with a common vocabulary, making it possible to interface phenotypic profiles of *OPA1* patients with those involving mutations in other genes or clinical presentations. It also contributes to a better understanding of polygenic diseases by connecting a patient to a large number of genes screened, as high-throughput sequencing now routinely allows, with each gene being validated by a specialized curator.

Finally, the database is directly queried by software suites dedicated to the annotation, filtering, and exploration of genomic variations, such as Alamut® (Interactive Biosoftware, France/SOPHiA GENETICS, Saint Sulpice, Switzerland). Thus, this open-access database should prove a valuable tool for clinicians and researchers alike.

## Methods

The original *e*OPA1 database published in 2005 [33] and updated in 2015 [35], was used as the starting point.

### Nomenclature

All names, symbols, and OMIM numbers were checked for correspondence with currently official names indicated by the Human Genome Organization (HUGO) Gene Nomenclature Committee [54] and the Online Mendelian Inheritance in Man database – OMIM® [55, 56]. The phenotype descriptions are based on HPO [43], indicating the HPO term identifier.

*OPA1* variants are described according to the *OPA1* transcript variant 8 (RefSeq: NM_130837.2), representing the longest transcript. Compared to transcript variant 1 (RefSeq: NM_015560.2), the original transcript identified, transcript variant 8, based on an alternate splice pattern characterized by Delettre et al. [11], contains two additional exons, 4b and 5b. However, it maintains the same reading frame encoding an isoform (8) of 1015 amino acids (aa). For standardization, the exons are numbered 1–30, instead of 1–4, 4b, 5, 5b, and 6–28, as originally proposed by Delettre et al. [11]. Furthermore, to maintain historical compatibility, variants are also described according to transcript variant 1 (when the mutation does not affect an alternative exon absent in variant 1). The numbering of the nucleotides reflects that of the cDNA, with “+ 1” corresponding to the “A” of the ATG translation initiation codon in the reference sequence, according to which the initiation codon is codon 1, as recommended by the version 2.0 nomenclature of the Human Genome Variation Society (HGVS): *http://varnomen.hgvs.org* [57].

Information concerning changes in RNA levels has been added from the original papers, or deduced from DNA if not experimentally studied. Following the HGVS guidelines, deduced changes are indicated between brackets.

### Implementation of the database

Our database has migrated to the “Global Variome shared Leiden Open-source Variation Database (LOVD)” currently running under LOVD v.3.0 Build 21 [58], following the guidelines for locus-specific databases (LSDBs) [59] and hosted under the responsibility of the Global Variome/Human Variome Project [42, 60].

The database for *OPA1* mutations includes a total of 21 items characterizing the DNA variants, 10 items characterizing the transcript variants (cDNA) (Fig. 1a), 7 items characterizing the molecular screenings (Fig. 1b), 14 items characterizing the individuals (Fig. 1c), and lastly, 24 items characterizing the phenotypes (Fig. 1d). A standardized description of the clinical and molecular items is set up using drop-down lists or list boxes with predefined variables. The clinical features are based on a large panel of symptoms encountered in ophthalmological, mitochondrial, and neurological diseases.

The *OPA1* database reviews clinical and molecular data from patients carrying *OPA1* variants published in peer-reviewed literature, as well as unpublished contributions that are directly submitted. While most variants can be described in terms of the latest update of the standard nomenclature, some inaccuracies may persist because gene anomalies discovered earlier might have been named according to a convention now out of use. Eventually, the “*DNA published*” field of the page dedicated to each variant (Fig. 1b) indicates whether the published name of the mutation has been modified by the curator. The *OPA1* LSDB website requires full compliance with the rules set out above for the description of sequence variants in order to provide uniform and comparable data.

### Data collection

The nomenclature of all causative variants in the *OPA1* database, published in 2015 [33], was reexamined. New causative variants were also searched for and collected from the literature published to date (October 12, 2018), using the NCBI PubMed search tool [61].

The positions of variants in the reference transcripts were determined and updated according to the HGVS nomenclature version 2.0 [57]. Correct naming at the nucleotide and amino acid levels were verified, and reestablished when necessary, using the Mutalyzer 2.0.28 *Syntax Checker* [62]. Exon numbering was updated with respect to the longest reference sequence (transcript variant 8) together with the originally identified reference sequence (transcript variant 1).

Information on the number of patients carrying each causative variant, as well as their geographical origins and the homo- or heterozygosity, was determined from the original or review papers, as well as from data collected during our local ophthalmology consultations. Further information on the genetic origin of the allele, segregation with the disease phenotype, and frequency in the control population was recorded. The results of functional studies were also incorporated.

The criteria of pathogenicity, which depend upon the clinical context and molecular findings, are stated under the headings: “*Affects function (as reported)”* for the pathogenicity as reported by the submitter, and “*Affects function (by curator)”* for the pathogenicity concluded upon by the curator (Fig. 1a). Putative novel variants detected in affected patients should segregate according to the disease status and not be present in control individuals. Putative variants are graded by the curator according to the type of mutation: frameshift and nonsense variants are considered to be pathogenic; missense variants are described as being of unknown pathogenicity when detected in single families without functional studies, or as probably pathogenic when detected in several families; the variants are considered to be pathogenic when so proven by experimental evidence or detected in multiple families. As new patients with existing variants are added to the database, the status of the variants is reassessed on the basis of the new data submitted.

### Data access and submission

The *OPA1* database is an open database allowing any researcher or clinician to consult the contents freely without prior registration, or to contribute new data after due registration to ensure traceability. The database can be accessed on the World Wide Web at: *https://www.lovd.nl/OPA1* (through the Global Variome shared LOVD server; or through the MITOchondrial DYNamics variation portal at: *http://opa1.mitodyn.org*). The data can also be retrieved via an application programming interface (API), i.e. a web service allowing simple queries and retrieval of basic gene and variant information (documentation available on the web page of the database); as well as serving as a public beacon in The Global Alliance for Genomics and Health Beacon Project [63].

General information is available at the database home page. The process for submitting data begins by clicking the “Submit” tab. Data discussed in this article is related to version OPA1:181012 (last updated on October 12, 2018). Data concerning new patients consulting at our Ophthalmological Center, added for this article since 2015, may be retrieved using the standard LOVD tabs (Individuals, Screenings, and Variants) by writing “Bastien Le Roux” in the “Owner” column. Data concerning new patients from the literature, added for this article since 2015, may be retrieved by writing “Thomas Foulonneau” in the “Owner” column. Data concerning new patients from our Molecular Genetics Laboratory, added for this article since 2015, may be retrieved by writing “Amati-Bonneau P” in the “Reference” column. Phenotypic data may be retrieved from the “Disease” tab by writing “OPA” and then following the link “Phenotype entries for this disease,” and again writing “Bastien Le Roux” or “Thomas Foulonneau”, respectively, as “Owner,” or “Marc Ferre” as “Owner”, and “> 0000143583” as “Phenotype ID.”

## Additional files

Additional file 1: Variants listed in the *OPA1* database that are unpublished in the literature (count: 149). (PDF 133 kb)

Additional file 2: Distribution of the 516 unique genomic variants in the LOVD *OPA1* database (full view). Eighteen large rearrangements (eleven deletions, six duplications, and one deletion-insertion) are shown as extended bars with rafters, substitutions as black bars, deletions as blue bars, insertions as green bars, and duplications as orange bars. At the top are reported the genomic coordinates on human chromosome 3 (assembly GRCh37/hg19), and *OPA1* transcript variants 1 and 8 structure in navy blue with alternative exons in pink, including exon numbering. Adapted from UCSC Genome Browser (*http://genome.ucsc.edu*) with the LOVD *OPA1* database custom track; data as of October 12, 2018. (PNG 1029 kb)

## Data Availability

The datasets generated and/or analyzed during the current study are available through the *OPA1* database, accessible in three different ways: (1) on the World Wide Web at: *https://www.lovd.nl/OPA1*; (2) via an application programming interface (API); and (3) as a public beacon in The Global Alliance for Genomics and Health Beacon Project [63].
