# Supplementary material for: OPA1: 516 unique variants and 831 patients registered in an updated centralized Variome database
Source: Orphanet J Rare Dis. 2019 Sep 10;14:214. doi: 10.1186/s13023-019-1187-1 (PMC6734442; doi:10.1186/s13023-019-1187-1)
Supplement: Supplementary file 1 — Variants listed in the OPA1 database that are unpublished in the literature (count: 149). (PDF 133 kb) [file 13023_2019_1187_MOESM1_ESM.pdf]

Additional file 1 Variants listed in the *OPA1* database that are unpublished in the literature (count: 149).

Exon/ Intron	DNA Variant[a]	RNA Variant[a]	Effect on Protein[a,b]	Classification of Variant[c]	Domain affected[d]	Database-ID[e]	Remarks
Exon 1	c.3G>A	r.?	p.?	Pathogenic	Basic	OPA1_000348	Variant in start codon
Exon 1	c.3G>T	r.?	p.?	Likely pathogenic	Basic	OPA1_000328	Variant in start codon
Exon 1	c.30del	r.(?)	p.(Cys11Valfs*7)	Pathogenic	Basic	OPA1_000462	
Intron 1-Intron 3	c.(32+1_33-1)_(448+1_449-1)del	r.spl	p.?	Likely pathogenic	Basic	OPA1_000349	Large deletion of exons 2–3[f]
Intron 1-Intron 29	c.(32+1_33-1)_(2983+1_2984-1)dup	r.?	p.?	Likely pathogenic	Basic, GTPase, Dynamin Central, Putative GED	OPA1_000273	Large duplication of exons 2–29[f]
Intron 1	c.32+24C>G	r.(?)	p.(=)	VUS	Basic	OPA1_000390	
Intron 1	c.32+87del	r.(?)	p.?	VUS	Basic	OPA1_000333	
Exon 2	c.43C>A	r.(?)	p.(Gln15Lys)	Benign	Basic	OPA1_000318	
Exon 2	c.50T>G	r.(?)	p.(Leu17*)	Pathogenic	Basic	OPA1_000465	
Exon 2	c.70A>G	r.(?)	p.(Ile24Val)	Likely benign	Basic	OPA1_000321	
Exon 2	c.108_125del	r.(?)	p.(Arg38_Ser43del)	VUS	Basic	OPA1_000397	
Exon 2	c.143T>G	r.(?)	p.(Leu48*)	Pathogenic	Basic	OPA1_000467	
Exon 2	c.224del	r.(?)	p.(Phe75Serfs*19)	Pathogenic	Basic	OPA1_000423	
Exon 2	c.245A>G	r.(?)	p.(Tyr82Cys)	Pathogenic	Basic	OPA1_000468	
Exon 2	c.267G>A	r.(?)	p.(Trp89*)	Pathogenic	Basic	OPA1_000327	
Exon 3	c.357del	r.(?)	p.(Phe119Leufs*7)	Pathogenic	Basic	OPA1_000323	
Exon 3	c.380C>T	r.(?)	p.(Pro127Leu)	VUS	Basic	OPA1_000389	
Exon 3	c.381G>A	r.(?)	p.(=)	Likely benign	Basic	OPA1_000336	
Exon 3	c.419del	r.(?)	p.(Val140Glyfs*24)	Pathogenic	Basic	OPA1_000469	
Exon 3	c.422G>A	r.(?)	p.(Trp141*)	Pathogenic	Basic	OPA1_000470	
Intron 3	c.448+1G>T	r.(spl?)	p.?	Pathogenic		OPA1_000418	
Intron 3-Intron 10	c.(448+1_449-1)_(1035+1_1036-1)dup	r.?	p.?	Likely pathogenic	GTPase	OPA1_000279	Large duplication of exons 4–10[f]
Exon 4	c.534A>G	r.(?)	p.(=)	Likely benign		OPA1_000341	
Exon 5	c.592G>A	r.(?)	p.(Asp198Asn)	VUS		OPA1_000398	
Intron 6	c.678+2T>G	r.spl	p.?	Likely pathogenic		OPA1_000330	
Intron 6	c.679-8C>T	r.(=)	p.(=)	Benign		OPA1_000399	
Exon 8	c.799A>T	r.(?)	p.(Lys267*)	Pathogenic		OPA1_000337	
Exon 8	c.814C>T	r.(?)	p.(Gln272*)	Pathogenic		OPA1_000320	
Exon 9	c.869G>A	r.(?)	p.(Arg290Gln)	VUS		OPA1_000400	
Exon 9	c.902T>G	r.(?)	p.(Leu301*)	Pathogenic		OPA1_000426	
Exon 9	c.910C>T	r.(?)	p.(Gln304*)	Pathogenic		OPA1_000419	
Exon 9	c.921_922del	r.(?)	p.(Asp307Glufs*6)	Pathogenic		OPA1_000472	
Intron 9	c.949-3C>A	r.(spl?)	p.?	VUS		OPA1_000325	
Exon 10	c.971C>T	r.(?)	p.(Ser324Phe)	Likely pathogenic	GTPase	OPA1_000272	
Exon 10	c.1017G>A	r.(=)	p.(=)	Benign	GTPase	OPA1_000401	
Exon 10	c.1018C>T	r.(?)	p.(Gln340*)	Pathogenic	GTPase	OPA1_000474	
Intron 10	c.1035+1del	r.spl	p.(=)	Pathogenic	GTPase	OPA1_000475	
Intron 10	c.1035+2T>A	r.spl	p.?	Likely pathogenic	GTPase	OPA1_000278	
Intron 10	c.1035+3A>C	r.(spl?)	p.(=)	Pathogenic	GTPase	OPA1_000477	

Intron 10	c.1035+20T>C	r.(=)	p.(=)	Likely benign	GTPase	OPA1_000403
Exon 11	c.1057A>G	r.(?)	p.(Ser353Gly)	Pathogenic	GTPase	OPA1_000306
Exon 11	c.1058G>A	r.(?)	p.(Ser353Asn)	Likely pathogenic	GTPase	OPA1_000332
Exon 11	c.1060G>C	r.(?)	p.(Ala354Pro)	Pathogenic	GTPase	OPA1_000376
Exon 11	c.1069A>C	r.(?)	p.(Thr357Pro)	VUS	GTPase	OPA1_000377
Exon 11	c.1113del	r.(?)	p.(Gly372Aspfs*5)	Pathogenic	GTPase	OPA1_000344
Exon 11	c.1130T>G	r.(?)	p.(Met377Arg)	VUS	GTPase	OPA1_000395
Intron 11	c.1149+1G>A	r.spl	p.?	Pathogenic	GTPase	OPA1_000310
Intron 11	c.1149+1G>C	r.spl	p.(=)	Pathogenic	GTPase	OPA1_000479
Exon 12	c.1174C>G	r.(?)	p.(His392Asp)	Likely pathogenic	GTPase	OPA1_000357
Exon 12	c.1181C>A	r.(?)	p.(Ala394Asp)	VUS	GTPase	OPA1_000314
Exon 12	c.1184T>C	r.(?)	p.(Leu395Pro)	VUS	GTPase	OPA1_000342
Exon 12	c.1200del	r.(?)	p.(Arg401Glyfs*14)	Pathogenic	GTPase	OPA1_000480
Intron 12	c.1230+2T>A	r.spl	p.?	Pathogenic	GTPase	OPA1_000335
Intron 12	c.1230+5G>A	r.(spl?)	p.?	Pathogenic	GTPase	OPA1_000392
Intron 12	c.1230+9A>G	r.(=)	p.(=)	VUS	GTPase	OPA1_000404
Exon 13	c.1231-15_1231-7delinsGCAG	r.(=)	p.(=)	Pathogenic	GTPase	OPA1_000427
Exon 13	c.1232_1233dup	r.(?)	p.(Ala412Leufs*4)	Pathogenic	GTPase	OPA1_000424
Exon 13	c.1262G>A	r.(?)	p.(Arg421Gln)	Likely pathogenic	GTPase	OPA1_000420
Exon 13	c.1302T>G	r.(?)	p.(=)	Likely benign	GTPase	OPA1_000329
Exon 13	c.1305G>C	r.(?)	p.(Glu435Asp)	Pathogenic	GTPase	OPA1_000481
Intron 13	c.1306-45C>T	r.(?)	p.(=)	Benign	GTPase	OPA1_000393
Exon 14	c.1324A>T	r.(?)	p.(Lys442*)	Pathogenic	GTPase	OPA1_000483
Exon 14	c.1342A>C	r.(?)	p.(=)	Likely benign	GTPase	OPA1_000319
Exon 14	c.1364C>T	r.(?)	p.(Pro455Leu)	Pathogenic	GTPase	OPA1_000486
Exon 14	c.1377del	r.(?)	p.(Asn459Lysfs*3)	Pathogenic	GTPase	OPA1_000313
Intron 14	c.1377+2dup	r.spl	p.?	Likely pathogenic	GTPase	OPA1_000354
Intron 14	c.1377+5G>A	r.(spl?)	p.(=)	VUS	GTPase	OPA1_000353
Intron 14	c.1378-7_1378-4del	r.(spl?)	p.?	VUS	GTPase	OPA1_000315
Exon 15	c.1379_1381delinsT	r.(?)	p.(Thr460Ilefs*8)	Pathogenic	GTPase	OPA1_000425
Exon 15	c.1391G>A	r.(?)	p.(Gly464Asp)	VUS	GTPase	OPA1_000340
Exon 15	c.1392dup	r.(?)	p.(Met465Hisfs*4)	Pathogenic	GTPase	OPA1_000489
Exon 15	c.1417del	r.(?)	p.(Ile473Phefs*49)	Pathogenic	GTPase	OPA1_000490
Exon 15	c.1429A>C	r.(?)	p.(Ser477Arg)	VUS	GTPase	OPA1_000384
Exon 15	c.1441A>T	r.(?)	p.(Met481Leu)	VUS	GTPase	OPA1_000405
Exon 15	c.1447A>C	r.(?)	p.(Asn483His)	Pathogenic	GTPase	OPA1_000492
Exon 16	c.1499G>A	r.(?)	p.(Arg500His)	Pathogenic	GTPase	OPA1_000104
Exon 16	c.1501A>T	r.(?)	p.(Ser501Cys)	VUS	GTPase	OPA1_000406
Exon 16	c.1534C>A	r.(?)	p.(Pro512Thr)	VUS	GTPase	OPA1_000383
Exon 16	c.1550C>T	r.(?)	p.(Thr517Ile)	Pathogenic	GTPase	OPA1_000495
Exon 16	c.1555_1557del	r.(?)	p.(Phe519del)	Pathogenic	GTPase	OPA1_000312
Exon 16	c.1577T>C	r.(?)	p.(Leu526Pro)	Pathogenic	GTPase	OPA1_000276
Exon 16	c.1599T>C	r.(=)	p.(=)	Likely benign	GTPase	OPA1_000407
Intron 16	c.1608+1G>T	r.(spl?)	p.?	Likely pathogenic	GTPase	OPA1_000538
Intron 16	c.1608+2T>C	r.spl	p.(=)	Pathogenic	GTPase	OPA1_000496

Exon 17	c.1609A>T	r.(spl?)	p.(Ile537Phe)	VUS	GTPase	OPA1_000385	
Exon 17	c.1643T>A	r.(?)	p.(Met548Lys)	VUS	GTPase	OPA1_000382	
Exon 17	c.1645A>T	r.(?)	p.(Lys549*)	Pathogenic	GTPase	OPA1_000421	
Exon 17	c.1667T>A	r.(?)	p.(Val556Asp)	Likely pathogenic	GTPase	OPA1_000338	
Intron 17	c.1682-16del	r.(?)	p.(=)	Likely benign		OPA1_000347	
Exon 18	c.1714_1715del	r.(?)	p.(Glu572Ilefs*2)	Pathogenic	Dynamin Central	OPA1_000497	
Exon 18	c.1714_1715dup	r.(?)	p.(Tyr573Asnfs*13)	Pathogenic	Dynamin Central	OPA1_000498	
Exon 18	c.1728_1734del	r.(?)	p.(Glu576fs)	Pathogenic	Dynamin Central	OPA1_000350	
Exon 18	c.1734del	r.(?)	p.(Gln579Argfs*6)	Pathogenic	Dynamin Central	OPA1_000499	
Intron 18	c.1754+1G>A	r.(?)	p.(=)	Pathogenic	Dynamin Central	OPA1_000501	
Intron 18	c.1754+2T>G	r.spl	p.(=)	Pathogenic	Dynamin Central	OPA1_000502	
Intron 18	c.1754+22T>G	r.(?)	p.(=)	VUS	Dynamin Central	OPA1_000379	
Intron 18	c.1755-75G>A	r.(?)	p.(=)	VUS	Dynamin Central	OPA1_000378	
Intron 18	c.1755-5A>T	r.(spl?)	p.?	Likely benign	Dynamin Central	OPA1_000428	
Exon 19	c.1806_1818del	r.(?)	p.(Val603Phefs*57)	Pathogenic	Dynamin Central	OPA1_000504	
Exon 19	c.1808dup	r.(?)	p.(Ser604Ilefs*13)	Pathogenic	Dynamin Central	OPA1_000386	
Intron 19	c.1870+1G>C	r.spl	p.(=)	Pathogenic	Dynamin Central	OPA1_000506	
Intron 19	c.1871-110C>T	r.(?)	p.(=)	Benign	Dynamin Central	OPA1_000380	
Exon 21	c.1939del	r.(?)	p.(Glu647Asnfs*17)	Pathogenic	Dynamin Central	OPA1_000508	
Exon 21	c.1966A>G	r.(?)	p.(Ile656Val)	VUS	Dynamin Central	OPA1_000429	
Exon 21	c.1975G>T	r.(?)	p.(Glu659*)	Pathogenic	Dynamin Central	OPA1_000509	
Intron 21-Intron 25	c.(2012+1_2013-1)_(2520+1_2521-1)dup	r.?	p.?	Likely pathogenic	Dynamin Central	OPA1_000355	Large duplication of exons 22–25[f]
Exon 22	c.2044del	r.(?)	p.(Arg682Glufs*6)	Pathogenic	Dynamin Central	OPA1_000511	
Exon 22	c.2089C>T	r.(?)	p.(Gln697*)	Pathogenic	Dynamin Central	OPA1_000513	
Exon 22	c.2109T>C	r.(=)	p.(=)	Likely benign	Dynamin Central	OPA1_000408	
Exon 22	c.2119A>G	r.(?)	p.(Thr707Ala)	Pathogenic	Dynamin Central	OPA1_000514	
Exon 22	c.2126A>G	r.(?)	p.(Asp709Gly)	Likely pathogenic	Dynamin Central	OPA1_000391	
Exon 22	c.2126del	r.(?)	p.(Asp709Valfs*17)	Pathogenic	Dynamin Central	OPA1_000515	
Exon 22	c.2139dup	r.(?)	p.(Gln714Thrfs*4)	Pathogenic	Dynamin Central	OPA1_000422	
Exon 22	c.2154_2157del	r.(?)	p.(Lys718Asnfs*7)	Pathogenic	Dynamin Central	OPA1_000387	
Exon 22	c.2155C>T	r.(?)	p.(Gln719*)	Pathogenic	Dynamin Central	OPA1_000316	
Exon 22	c.2162del	r.(?)	p.(Pro721Leufs*5)	Pathogenic	Dynamin Central	OPA1_000516	
Exon 23	c.2213G>A	r.(?)	p.(Arg738His)	VUS	Dynamin Central	OPA1_000409	
Exon 23	c.2262del	r.(?)	p.(Lys754Asnfs*24)	Pathogenic	Dynamin Central	OPA1_000518	
Exon 23	c.2267_2268del	r.(?)	p.(Lys756Argfs*4)	Pathogenic	Dynamin Central	OPA1_000326	
Exon 23	c.2313C>G	r.(?)	p.(Asp771Glu)	Benign	Dynamin Central	OPA1_000410	
Exon 24	c.2338_2339del	r.(?)	p.(Ile780Serfs*12)	Pathogenic	Dynamin Central	OPA1_000522	
Intron 24	c.2441-13T>G	r.(?)	p.?	VUS	Dynamin Central	OPA1_000324	
Exon 25	c.2459dup	r.(?)	p.(Asn820Lysfs*26)	Pathogenic	Dynamin Central	OPA1_000331	
Exon 25	c.2475C>G	r.(?)	p.(Asp825Glu)	VUS	Dynamin Central	OPA1_000351	

Exon 25	c.2513A>G	r.(?)	p.(Gln838Arg)	Pathogenic	Dynamin Central	OPA1_000524	
Intron 25	c.2520+2T>C	r.(spl?)	p.?	Pathogenic	Dynamin Central	OPA1_000411	
Intron 25	c.2521-1G>T	r.spl	p.?	Pathogenic	Dynamin Central	OPA1_000343	
Exon 26	c.2619del	r.(?)	p.(Lys874Argfs*8)	Pathogenic	Dynamin Central	OPA1_000525	
Exon 26	c.2657G>A	r.(?)	p.(Ser886Asn)	VUS	Dynamin Central	OPA1_000381	
Exon 26	c.2661G>A	r.(?)	p.(=)	Pathogenic	Dynamin Central	OPA1_000526	
Exon 27	c.2694A>T	r.(?)	p.(Arg898Ser)	VUS		OPA1_000412	
Exon 27	c.2709A>G	r.(=)	p.(=)	Benign		OPA1_000413	
Intron 27	c.2779-1G>T	r.spl	p.?	Pathogenic		OPA1_000345	
Exon 28	c.2796_2798del	r.(?)	p.(Val933del)	VUS		OPA1_000274	
Exon 28	c.2806dup	r.(?)	p.(Trp936fs)	Pathogenic		OPA1_000275	
Exon 28	c.2809C>G	r.(?)	p.(Arg937Gly)	VUS		OPA1_000414	
Intron 28	c.2873-163G>A	r.(?)	p.(=)	Benign		OPA1_000388	
Intron 28	c.2873-2_2874del	r.?	p.?	Pathogenic		OPA1_000415	
Exon 29	c.2884G>T	r.(?)	p.(Glu962*)	Pathogenic	Putative GED	OPA1_000339	
Exon 29	c.2890_2891insTTA	r.(?)	p.(Lys963_Asn964insIle)	VUS	Putative GED	OPA1_000334	
Exon 29	c.2899G>T	r.(?)	p.(Glu967*)	Pathogenic	Putative GED	OPA1_000533	
Exon 29	c.2948T>C	r.(?)	p.(Leu983Pro)	Pathogenic	Putative GED	OPA1_000534	
Exon 29	c.2983A>T	r.(?)	p.(Lys995*)	Pathogenic	Putative GED	OPA1_000535	
Exon 29-Intron 29	c.2983_2983+1del	r.spl	p.?	Pathogenic	Putative GED	OPA1_000317	
Intron 29-Intron 31	c.(2983+1_2984-1)_(*20_?)del	r.spl	p.?	Pathogenic	Putative GED	OPA1_000346	Large deletion of exons 30–31[f]
Intron 29	c.2984-4A>G	r.(spl?)	p.?	Benign	Putative GED	OPA1_000416	
Exon 30	c.2988dup	r.(?)	p.(Val997Serfs*2)	Pathogenic	Putative GED	OPA1_000305	
Exon 30	c.3001C>T	r.(?)	p.(Gln1001*)	Pathogenic	Putative GED	OPA1_000352	
Exon 30	c.3010C>A	r.(?)	p.(Leu1004Ile)	VUS	Putative GED	OPA1_000417	

Note: Data as of October 12, 2018; for more information, please refer to the database using Database-ID.

[a]Mutational data are described using the nomenclature of the Human Genome Variation Society (http://www.hgvs.org/mutnomen). Nucleotide numbering reflects cDNA numbering with "+1" corresponding to the A of the ATG translation initiation codon in the reference sequence (*OPA1* transcript variant 8, RefSeq: NM_130837.2), according to journal guidelines. The initiation codon is codon 1.

[b]Predicted effect on protein based on clinical consequences

[c]Classification of variant based on clinical consequences, using standardized criteria: pathogenic (disease-associated), likely pathogenic (likely disease-associated), VUS (variant of unknown significance), likely benign (likely not disease-associated), benign (not disease-associated).

[d]Affected domain of the protein: basic domain (mitochondrial target peptide, exons 1–3), GTPase domain (exons 10–17), dynamin central region (exons 18–26), region probably corresponding to a GTPase effector domain (GED, exons 29–30).

[e]Identifier of variant in the *OPA1* database (*https://www.lovd.nl/OPA1*).

[f]Break points have not been fully characterized
