# Supplementary figures and images for: OPA1: 516 unique variants and 831 patients registered in an updated centralized Variome database
Source: Orphanet J Rare Dis. 2019 Sep 10;14:214. doi: 10.1186/s13023-019-1187-1 (PMC6734442; doi:10.1186/s13023-019-1187-1)

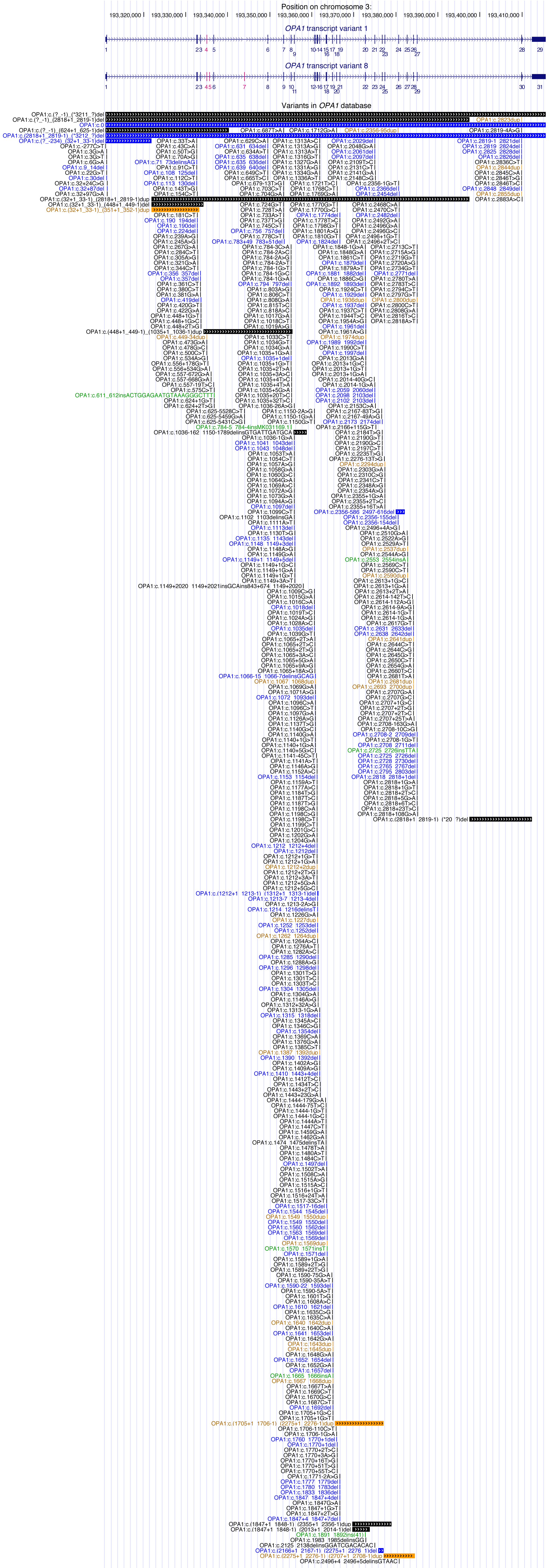

Supplement: Supplementary file 2 — Distribution of the 516 unique genomic variants in the LOVD OPA1 database (full view). Eighteen large rearrangements (eleven deletions, six duplications, and one deletion-insertion) are shown as extended bars with rafters, substitutions as black bars, deletions as blue bars, insertions as green bars, and duplications as orange bars. At the top are reported the genomic coordinates on human chromosome 3 (assembly GRCh37/hg19), and OPA1 transcript variants 1 and 8 structure in navy blue with alternative exons in pink, including exon numbering. Adapted from UCSC Genome Browser (http://genome.ucsc.edu) with the LOVD OPA1 database custom track; data as of October 12, 2018. (PNG 1029 kb) [file 13023_2019_1187_MOESM2_ESM.png]
